# Antioxidant and Antifungal Effects of Six Plant Essential Oils Against *Penicillium digitatum* and *Penicillium italicum*

**DOI:** 10.3390/microorganisms13092042

**Published:** 2025-09-01

**Authors:** María del Carmen García-Custodio, Francisco Márquez-García, David García-Alonso, Cristian David Brieva-Trejo, Francisco María Vázquez Pardo

**Affiliations:** Centro de Investigaciones Científicas y Tecnológicas de Extremadura (CICYTEX), Instituto de Investigaciones Agrarias Finca La Orden-Valdesequera, Área de Biodiversidad Vegetal Agraria, Autovía A-5 Km 372, Guadajira, 06187 Badajoz, Spain; carmen.garciac@juntaex.es (M.d.C.G.-C.); frvazquez50@hotmail.com (F.M.V.P.)

**Keywords:** antifungal activity, antioxidant, essential oil, lavandula, mentha, origanum, penicillium, thymus

## Abstract

Six aromatic plants (*Lavandula pedunculata* subsp. *sampaioana*, *Lavandula stoechas* subsp. *luisieri*, *Mentha × piperita*, *Origanum vulgare* subsp. *virens*, *Thymus mastichina*, and *Thymus zygis* subsp. *sylvestris*) were analyzed to evaluate their essential oil (EO) yield, chemical composition, antioxidant activity, and antifungal capacity against two mold species, green mold (*Penicillium digitatum*) and blue mold (*Penicillium italicum*). The antioxidant activity was evaluated using the ABTS and DPPH methods, and the antifungal activity was determined using the disk diffusion method. The results of the antioxidant activity tests showed that the essential oil of *Th. zygis* subsp. *sylvestris* has the highest value for the ABTS method (161.70 ± 0.15 mM TROLOX eq. and 864.20 ± 0.81 g TROLOX eq/g EO) and the *L. stoechas* subsp. *luisieri* essential oil in the DPPH method (33.91 ± 1.21 mM TROLOX eq. and 184.99 ± 6.58 g TROLOX eq/g EO). Furthermore, the essential oils with lower antioxidant activity were *L. pedunculata* subsp. *sampaioana* for the ABTS method (3.84 ± 0.26 mM TROLOX eq. and 20.79 ± 1.41 g TROLOX eq/g EO) and *Th. mastichina* for DPPH method (0.96 ± 0.03 mM TROLOX eq. and 5.31 ± 0.16 g TROLOX eq/g EO). *Th. zygis* subsp. *sylvestris* exhibited the strongest antifungal activity, with medium inhibition halo values of 60.50 ± 5.77 mm and 54.33 ± 2.93 mm for *P. digitatum* and *P. italicum*, respectively.

## 1. Introduction

Postharvest fruit and vegetable diseases caused by fungal infections (*Aspergillus*, *Penicillium*, *Fusarium*, *Alternaria*, …) due to wounds or insect bites produce an elevated loss of food during storage, distribution, and sale [1,2,3,4,5,6,7]. Citrus major sources of postharvest diseases are green mold (*Penicillium digitatum* (Pers.) Sacc.) and blue mold (*Penicillium italicum* Wehmer), which cause economic losses of 15–30% and affect 50–90% of production, particularly in developing countries [8,9,10,11,12,13]. Traditionally, several methods based on synthetic chemical fungicides have been developed to reduce post-harvest losses; however, the intensive use of these methods generates resistance, reducing their effectiveness [9,14,15]. Moreover, consumer trends demand products that are free of chemical residues and more environmentally friendly. Together with legislative restrictions on the use of phytosanitary products, this creates the need for new, more effective and environmentally friendly postharvest management. These alternatives include biocontrol strategies involving the use of antagonist yeast or bacteria, immersion in aqueous extracts of medicinal plants or citrus fruits, vaporization of essential oils from medicinal plants, wax coatings containing essential oils or plant extracts, new biopolymers and heat treatments, among others [6,11,16,17,18,19,20,21].

Essential oils and medicinal plant extracts are composed of mixtures of volatile organic compounds. These include alcohols, ethers, oxides, aldehydes, ketones, esters, amines, amides, phenols and heterocycles, as well as mainly terpenes [22,23]. The presence of these compounds varies depending on the plant species and, within the same species, the percentage of each chemical compound is influenced by the plant’s geographical origin, environmental conditions, and the storage process of the plant material and essential oil [24,25,26,27]. Therefore, it is crucial to understand the chemical composition of any essential oil, regardless of the plant species it comes from.

Work is currently underway to identify active ingredients of natural origin that are more environmentally friendly to produce and use. The aim is to develop effective treatments for antibiotic resistance in human and animal health and herbicides for agricultural crops [28,29,30,31]. This has prompted research into the chemical composition of essential oils, their antifungal, antibacterial, and antimicrobial properties, and their potential use in treating respiratory diseases, etc. [32,33,34,35,36].

Studies of the antifungal capacity of medicinal plants extracts or essential oils have reported the ability of fight various fungal infections caused by *Aspergillus* spp., *Candida* spp., *Cryptococcus* spp., *Epidermophyton* spp., *Fusarium* spp., *Microsporum* spp. *Penicillium* spp., and *Trichophyton* spp. [37,38,39,40,41]. Several studies have shown that essential oils from species such thyme, oregano, clove, cinnamon or citrus have a high inhibitory capacity against the in vitro growth of fungal colonies from *Penicillium* species such as *P. digitatum* and *P. italicum*) [42,43,44,45,46,47,48,49,50]. The antifungal properties of these essential oils have contributed to the development of new research aimed at preventing post-harvest infections caused by *P. digitatum* and *P. italicum* [51,52,53,54,55,56,57,58,59].

The use of essential oils from medicinal plants whose chemical composition includes antifungal compounds (e.g., thymol, carvacrol, terpinen-4-ol, etc. [59]) makes it possible to search for local medicinal species commonly used in traditional medicine that are rich in these kinds of compounds, creating a new local employment opportunity that are more environmentally friendly. For that purpose, the main objective of this research is to evaluate the inhibitory and antioxidant activities of different aromatic plants, native to the SW from the Iberian Peninsula, against two mold species (*P. digitatum* and *P. italicum*), which cause postharvest disease in citrus fruits (e.g., oranges).

## 2. Materials and Methods

### 2.1. Plant Material, Essential Oil Extraction, and Chemical Characterization of Essential Oils

Aerial parts of six aromatic plants (*Lavandula pedunculata* subsp. *sampaioana* (Rozeira) Franco, *Lavandula stoechas* subsp. *luisieri* (Rozeira) Rozeira, *Mentha × piperita* L., *Origanum vulgare* subsp. *virens* (Hoffmans. & Link) Bonnier & Layens, *Thymus mastichina* (L.) L., and *Thymus zygis* subsp. *sylvestris* (Hoffmanns. & Link) Cout.) were collected from the experimental crops at Institute of Agrarian Research “La Orden-Valdesequera” (CICYTEX) (near of Guadajira, Spain). Representative samples were collected during the flowering stage, which took place between May and June 2024.

Fresh stems, leaves, and flowers from each specie were cut into small pieces and submitted to hydro-distillation in Clevenger-type apparatus for 2 h. The essential oils (EOs) were stored in amber vial at 4 °C.

The chemical analysis of the essential oils was carried out using a combination of two gas chromatography techniques (GC-FID + GC-MS), chemical compounds were identified by CG-MS and quantified by CG-FID. The analysis was performed on Agilent 8890 GC paired with the 5977B MSD (Mass Selective Detector). Polar column DB-WAX UI (60 m long, 0.25 mm diameter and 0.5 µm film thicknesses) was employed using Helium carrier gas at constant flow of 2 mL/min. Apolar column HP-5MS UI (60 m long, 0.25 mm diameter and 0.25 µm film thicknesses) was employed using Helium carrier gas at constant flow of 1 mL/min. The column temperature started at 50 °C and increased to 240 °C (polar column) and 285 °C (apolar column).

### 2.2. Antioxidant Activity

The antioxidant activity of each essential oil sample was determined by ABTS and DPPH assay method. The absorbance was measured using a spectrophotometer (Beckman Coulter DU^®^ 730, Beckman Coulter, Inc., Brea, CA, USA).

The standard line from each assay was designed using Trolox (6-hidroxy-2,5,7,8-tetramethylchroman-carboxylic acid) (Sigma-Aldrich 238813, Sigma-Aldrich, Inc., St. Louis, MO, USA) between 1 mM and 2 mM concentration and measured the absorbance at 734 nm (ABTS) and 517 nm (DPPH).

All the essential oil samples were analyzed in triplicate. The sample volume used was 3 mL (2.95 mL from DPPH/ABTS + 50 μL from essential oil sample). The results, from both analyses (ABTS and DPPH) were presented as millimoles (mM) of Trolox equivalents and grams of Trolox equivalents per gram of essential oil, with the main objective of developing a data matrix comparable between each other.

#### 2.2.1. ABTS [2,2′-Azino-bis(3-ethylbenzothiazoline-6-sulphonic acid)] Assay

ABTS assay is based on the ability of molecules to scavenge the free radical of ABTS in comparison with Trolox [60]. Absolute ethanol was used to prepare the working solution of ABTS (Sigma-Aldrich A1888) at a concentration of 7 mM, which was then adjusted to obtain a final absorbance of 0.7 ± 0.02 (at 734 nm). To determine antioxidant activity, the essential oil samples remained in the dark at ambient temperature for 30 min and, thereafter, the absorbance was measured at 734 nm.

#### 2.2.2. DPPH (2,2-Diphenyl-1-picrylhydrazyl) Assay

The DPPH protocol to measure antioxidant activity was based on the description in reference [61]. Methanol (100%) was used as the solvent to prepare a working solution 75 µmol/L of DPPH (Sigma-Aldrich D9132), which was then adjusted to a final absorbance of 0.7 ± 0.02 (at 517 nm). For antioxidant activity determination, the samples remained in the dark at an ambient temperature for 120 min, after which the absorbance was measured at 517 nm.

### 2.3. In Vitro Antifungal Activity Assay

#### 2.3.1. Fungal Isolation

The fungal species used are *P. digitatum* and *P. italicum*, obtained from infected *Citrus aurantium* L. fruit. The isolation was realized in Petri dishes containing Sabouraud Dextrose agar (6%) and incubated for 7 days at 27 °C ± 1 °C, in complete darkness. The differential isolations were transferred to new Petri dishes containing Sabouraud Dextrose agar and re-sown each week until a pure fungal culture of each species was obtained. Finally, the standardization of the fungal colonies was achieved using a 0.85% saline solution suspension to obtain the 0.5 McFarland standard (1.5 × 10^8^ CFU/mL) [62]. Morphological characterization (macro and microscopic) was performed using dichotomous keys as a reference [63,64,65].

#### 2.3.2. Antifungal Activity–Disk Diffusion Method

The disk diffusion method was used to evaluate the antifungal activity of each of the essential oils [66,67]. The fungal suspension was sown in Petri dishes (90 mm diameter) containing 25 mL of Sabouraud Dextrose Agar. A sterile swab was used to spread the mold suspension (1.5 × 10^8^ CFU/mL) evenly across the surface of the dish to ensure a homogeneous development of the mold. Essential oils were inoculated using a 10 mm diameter filter disk soaked with 25 μL of each essential oil sample and placed in the center of the Petri dish. A control group was included in the study to ensure that the mold growth was unaffected by the essential oil. The control group was processed in the same way as the study group, but with distilled water instead of essential oil.

The study included a control group and a study group with 3 repetitions of each for each species of essential oil. Thus, 12 Petri dishes were used for each essential oil species (3 control dishes + 3 study dishes for each one of the analyzed molds, *P. digitatum* and *P. italicum*). The Petri dishes were incubated for 5 days (96 h) in an incubator chamber at 27 °C ± 1 °C in complete darkness and in the normal position (not inverted) to avoid affecting the mold growth. Finally, the Petri dishes were checked, photographed, and measured every 24 h. Measurements were taken by evaluating the inhibitory halo of growth around the filter disk using a caliper.

### 2.4. Statistical Analysis

Descriptive and inferential statistical analysis were performed using R v 4.3.3 software [68] to determine the relationship between the inhibitory halo of growth results and the antioxidant activity obtained from the samples. The 48 h data from the inhibitory halo of growth were used to develop statistical analysis (to ensure a correct understanding of the data and avoid mixing up inhibition and natural absence of growth). This also ensures the correct effect of the essential oil and prevents it from evaporating during the process.

## 3. Results

### 3.1. Essential Oil Composition

Table 1 shows the essential oil yield obtained for each aromatic plant, expressed in grams of essential oil per kilogram of fresh plant and as a percentage (*w*/*w*). The highest yields were obtained in *Th. mastichina*, *L. pedunculata* subsp. *sampaioana*, and *Th. zygis* subsp. *sylvestris* with values of 2.43%, 1.28%, and 0.88%, respectively. The species with the lowest yields were *M. × piperita*, *L. stoechas* subsp. *luisieri*, and *O. vulgare* subsp. *virens* (0.62%, 0.42% and 0.41%, respectively).

The essential oils have a rich monoterpene-based chemical composition (Table 2). The majority of the detected compounds are: thymol (68.83% in *Th*. *zygis* subsp. *sylvestris* and 36.72% in *O. vulgare* subsp. *virens*), 1.8-cineole (66.06% and 17.71% in *Th. mastichina* and *L. stoechas* subsp. *luisieri*, respectively), camphor and fenchone (35.51% and 34.20%, respectively, in *L. pedunculata* subsp. *sampaioana*), gamma-terpinene (30.69% in *O. vulgare* subsp. *virens*), menthone and L-menthol (29.12% and 27.56%, respectively, in *M. × piperita*), and trans-alpha-necrodyl acetate (20.46% in *L. stoechas* subsp. *luisieri*).

### 3.2. Antioxidant Activity

The obtained results (Table 3) show that the essential oils of the *L. stoechas* subsp. *luisieri*, *O*. *vulgare* subsp. *Virens*, and *Th*. *zygis* subsp. *sylvestris* species have higher antioxidant activity. These species have in common a high percentage of the chemical’s thymol, gamma-terpinene and trans-alpha-necrodyl acetate in their essential oils.

The essential oils tested showed a higher antioxidant capacity using the ABTS method than the DPPH method. In the ABTS method, the essential oils with the highest antioxidant capacity values are as follows: *Th. zygis* subsp. *sylvestris* (161.70 ± 0.15 mM TROLOX eq.), *O. vulgare* subsp. *virens* (76.45 ± 3.02 mM TROLOX eq.), and *L. stoechas* subsp. *luisieri* (24.06 ± 0.64 mM TROLOX eq.). By contrast, the results obtained using the DPPH method show a smaller difference between the essential oils with the highest antioxidant capacity belong to the same species of medicinal plants: *L. stoechas* subsp. *luisieri* (33.91 ± 1.21 mM TROLOX eq.), *Th. zygis* subsp. *sylvestris* (25.34 ± 1.08 mM TROLOX eq.), and *O. vulgare* subsp. *virens* (25.15 ± 1.69 mM TROLOX eq.).

In contrast, the species *Th. mastichina*, *L. pedunculata* subsp. *sampaioana*, and *M. × piperita* exhibited low antioxidant activity values using both methods, with higher values obtained using the ABTS than the DPPH method. The essential oil of *Th. mastichina* (0.96 ± 0.03 mM TROLOX eq.) exhibited the lowest antioxidant activity value in the DPPH method, followed by *L. pedunculata* subsp. *sampaioana* and *M. × piperita* (2.17 ± 0.16 mM TROLOX eq. and 3.83 ± 0.13 mM TROLOX eq., respectively). In the ABTS method, the essential oils of *L. pedunculata* subsp. *sampaioana* and *M. × piperita* have the lowest antioxidant capacity (3.84 ± 0.26 mM TROLOX eq. and 4.83 ± 0.09 mM TROLOX eq., respectively), whereas *Th. mastichina*. has a greater reducing capacity in this method than in the DPPH method (9.76 ± 0.41 mM TROLOX eq.).

### 3.3. In Vitro Antifungal Activity Assay

#### 3.3.1. Fungal Isolation

**Figure 1:**
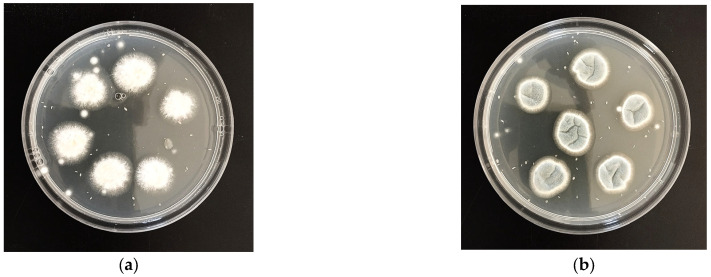
Fungal isolation: (**a**) *P. digitatum*; (**b**) *P. italicum*.

The *P. italicum* species was observed to grow more quickly in in vitro conditions than *P. digitatum* (Figure 1).

#### 3.3.2. Antifungal Activity

**Figure 2:**
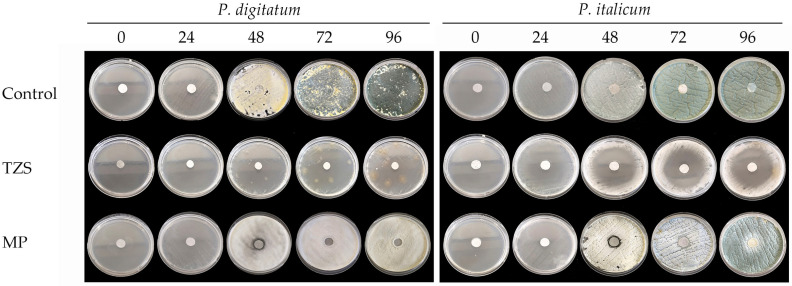
Photographic progression of *P. digitatum* and *P*. *italicum* (time in hours). (Note: TZS: *Th. zygis* subsp. *sylvestris*, MP: *M. × piperita*).

The Petri dishes were photographed every 24 h, and it could be observed that the filter disk infused with the essential oils is able to inhibit the fungal species development (inhibition halo) and delay conidiospore development of both species. Figure 2 shows an example of the essential oil effects with different antifungal effects: *Th. zygis* subsp. *sylvestris* (high antifungal effects) and *M*. × *piperita* (low antifungal effects), both compared with the control group.

Table 4 displays statistical parameters from the inhibition halo measurements obtained after 48 h of growth (the 48 h data were used to ensure a correct understanding of the data and avoid mixing up inhibition and natural absence of growth as well as to ensure a correct effect of the essential oil and avoid the evaporation of it during the process). Measurement results show a higher inhibitory capacity from essential oil over *P. digitatum* species than *P. italicum*. 

**Figure 3:**
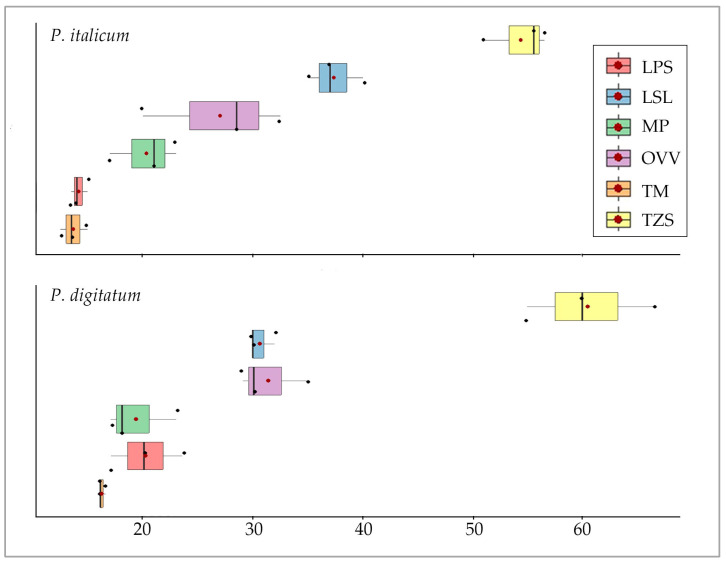
Inhibition halo boxplot produced by the different essential oil samples. (Note 2: OVV: *O*. *vulgare* subsp. *virens*, LPS: *L*. *pedunculata* subsp. *sampaioana*, LSL: *L*. *stoechas* subsp. *luisieri*, TM: *Th*. *mastichina*, TZS: *Th*. *zygis* subsp. *sylvestris*, MP: *M*. *× piperita*).

Finally, the data distribution in relation to each essential oil species is presented in a boxplot for each fungal species (Figure 3). It is possible to observe that the essential oil of *Th. zygis* subsp. *sylvestris* produced the largest inhibition halo (60.50 ± 5.77 mm and 54.33 ± 2.93 mm for *P. digitatum* and *P. italicum*, respectively), with a clear difference compared to the rest of the samples. The next species showing the highest antifungal activity were *L. stoechas* subsp. *luisieri* (30.67 ± 1.15 mm and 37.33 ± 2.52 mm for *P. digitatum* and *P. italicum*, respectively) and *O. vulgare* subsp. *virens* (31.33 ± 3.21 mm and 27.00 ± 6.38 mm for *P. digitatum* and *P. italicum*, respectively).

The essential oil of *Th. mastichina* demonstrated the lowest level of antifungal activity, exhibiting an inhibition halo of 13.67 ± 1.26 mm over *P. italicum* y 16.17 ± 0.29 mm over *P. digitatum*.

## 4. Discussion

The obtained data shows the essential oils of *Th. zygis* subsp. *sylvestris*, *O. vulgare* subsp. *virens* and *L. stoechas* subsp. *luisieri* have high antioxidant activity for both ABTS and DPPH and elevated antifungal activity. Differences are observed in the results for each essential oil when using the ABTS and DPPH methods. This is due to the different reduction mechanisms used by each method: DPPH is based on proton elimination, while ABTS is based on proton interchange [69]. Conversely, the essential oils of *Th*. *mastichina*, *M. × piperita* and *L. pedunculata* subsp. *sampaioana* exhibited low antioxidant and antifungal activity against the two evaluated fungal species, *P. italicum* and *P. digitatum*.

In terms of their chemical composition, the essential oils of *Th. zygis* subsp. *sylvestris* and *Origanum vulgare* subsp. *virens* are rich in thymol (monoterpene phenol), while *L. stoechas* subsp. *luisieri* contains trans-alpha-necrodyl acetate (monoterpene ester). *L. pedunculata* subsp. *sampaioana* contains mainly fenchone and camphene (monoterpene ketones). Finally, *Th. mastichina* and *M. × piperita* contain mainly 1,8-cineole (a monoterpene ether), menthone (monoterpenic ketone), and menthol (monoterpene alcohol), respectively. Correlating the main component of each essential oil with its antioxidant and antifungal properties shows that phenolic monoterpenes and esters are more inhibitory than alcohol, ketone and ether monoterpenes.

High antifungal and antioxidant activity from *Th. zygis* has been widely recognized in several studies [70,71,72,73,74,75]. *Th. zygis* essential oil can have different chemotypes (thymol, carvacrol, carvacrol/thymol, linalool, geranyl acetate/geraniol, …) [72,74,75,76,77]. However, only the carvacrol, thymol, and carvacrol/thymol chemotypes, have demonstrated elevated antifungal and antioxidant capacity [60,62,63,64,65,66]. The presence of thymol and carvacrol compounds in essential oil from other species of *Thymus* L. genus is well known [47,78,79,80]. Furthermore, research into the antifungal activity indicates that they have a higher inhibitory capacity for fungal growth than the pure compounds thymol or carvacrol [71,78]. Regarding *P*. *digitatum* and *P*. *italicum* molds, *Th*. *zygis* subsp. *sylvestris* essential oil has an elevated inhibitory capacity against “in vitro” growth, as observed in other *Penicillium* species [47,50,70].

On the other hand, the other thyme species included in this study, *Th. mastichina*, has an essential oil rich in 1,8-cineole [73,81,82,83], with a poor antioxidant and antifungal capacity against the *Penicillium* species studied. However, other studies have shown it to have good antifungal properties against other fungal species, such as *Sclerotinia* spp., *Fusarium* spp., *Alternaria* spp. or *Candida* spp. [73,84,85,86]. This makes it possible to use it to fight fungal infections in crops or on the skin.

*O. vulgare* subsp. *virens* essential oil has a thymol/gamma-terpinene chemotype, which is unusual for this species [87,88]. This coincides with what was observed in research involving the carvacrol chemotype of *O. vulgare* subsp. *vulgare*, which exhibits high antioxidant and antifungal activity against *P. digitatum* and *P. italicum* [50,88,89,90,91].

The two *Lavandula* L. subspecies studied exhibit different antifungal capacities, with *L. stoechas* subsp. *luisieri* demonstrating greater activity than *L. pedunculata* subsp. *sampaioana* [92], and notably the inhibitory effect on *P. digitatum* growth is higher than on *P. italicum*. The antioxidant activity of *L. stoechas* subsp. *luisieri* essential oil is very high, mainly due to the presence of necrodiol derivatives [93]. On the other hand, *L. pedunculata* subsp. *sampaioana* has an essential oil rich in fenchone, camphor and 1,8-cineole, which are compounds with low antioxidant capacity [73,82].

*M. × piperita* essential oil exhibits the lowest of all the essential oils studied in the present research. However, other studies indicate good inhibitory capacity against several species of the *Penicillium* genus, including *P. digitatum* [94,95,96,97]. This divergence in results could be due to variation in the essential oil’s chemical composition, including different percentages of menthol, menthone, limonene, alpha-pinene, and betha-pinene, among others.

## 5. Conclusions

The *Th. zygis* subsp. *sylvestris*, *O. vulgare* subsp. *Virens*, and *L. stoechas* subsp. *luisieri* essential oils have a high antioxidant capacity and can effectively inhibit the “in vitro” growth of the molds (*P. digitatum* and *P. italicum*) that mainly cause postharvest damages in *Citrus* genus fruits. Furthermore, all the essential oils studied exhibited a higher inhibition response against green mold (*P. digitatum*) than blue mold (*P. italicum*).

Essential oils with a chemical composition rich in phenolic monoterpenes and ethers have greater antioxidant and antifungal capacity against *P. digitatum* and *P. italicum* molds, as they inhibit spore germination for a longer period of time.

## Figures and Tables

**Table 1:** Yield of the essential oil extraction by hydrodistillation.

Species	Code	Yield (*w*/*w*) (g/kg)	% (*w*/*w*)
*O. vulgare* subsp. *virens*	OVV	4.09	0.41
*L. pedunculata* subsp. *sampaioana*	LPS	12.80	1.28
*L. stoechas* subsp. *luisieri*	LSL	4.22	0.42
*Th. zygis* subsp. *sylvestris*	TZS	8.78	0.88
*Th. mastichina*	TM	24.29	2.43
*M*. × *piperita*	MP	6.17	0.62

**Table 2:** Composition of the essential oils. (Note 1: value in %). (Note 2: OVV: *O*. *vulgare* subsp. *virens*, LPS: *L*. *pedunculata* subsp. *sampaioana*, LSL: *L*. *stoechas* subsp. *luisieri*, TM: *Th*. *mastichina*, TZS: *Th*. *zygis* subsp. *sylvestris*, MP: *M*. *× piperita*).

RI-WAX	RI-HP5	Compound	OVV	LPS	LSL	TM	TZS	MP
1025	933	Alpha-Pinene	0.67	6.35	1.83	3.44	0.51	0.74
1029	918	Alpha-Thujene	1.63	0.01		0.21	1.32	0.06
1069	953	Camphene	0.29	2.29	0.10	0.10	0.14	0.02
1114	978	Beta-Pinene	0.18	0.05	0.30	5.11	0.13	1.17
1126	972	Sabinene	0.29	0.03	0.12	3.83	0.07	0.67
1133	940	Cymene Isomer			2.67			
1165	991	Beta-Myrcene	2.28	0.17	0.07	1.87	1.91	0.33
1186	1018	Alpha-Terpinene	3.76	0.03			1.33	0.26
1206	1021	Limonene	0.35	2.03	0.20	1.17	0.32	3.42
1222	1039	1,8-Cineole	0.02	0.93	17.71	66.06		6.72
1238	1035	Cis-Beta-Ocimene	2.15	0.15	0.45	0.02	0.01	0.27
1254	1058	Gamma-Terpinene	30.69	0.05	0.12	1.79	5.72	0.41
1278	1025	Para-Cymene	5.26	0.24	0.14	1.03	9.36	0.08
1418	1090	Fenchone		34.20	0.28			
1465	1074	Trans-Sabinene Hydrate	0.16			0.70	0.68	0.82
1484	1124	Menthone						29.12
1500	1164	Menthofuran						4.94
1510	1166	Isomenthone						4.31
1541	1149	Camphor		36.51	1.00			
1553	1100	Linalool	0.16	2.00	2.29	4.14	0.86	0.26
1574	1294	Menthyl Acetate						2.36
1592	1239	Thymol Methyl Ether	1.88				0.01	
1595	1119	Fenchol <endo->		0.86				
1599	1288	Bornyl Acetate		0.98	0.06	<0.01		
1606	1280	Trans-Alpha-Necrodyl Acetate			20.46			
1607	1239	Carvacrol Methyl Ether	2.38				0.05	
1608	1165	Neo-Menthol						4.11
1617	1450	Trans-Beta Caryophyllene	1.61	0.04	0.22	0.12	1.41	1.18
1619	1284	Lavandulyl Acetate		0.20	4.01			
1636	1296	Arbozol			2.24			
1653	1169	L-Menthol						27.56
1662	1170	Delta-Terpineol				1.50		0.22
1665	1244	Pulegone						3.98
1668	1187	5-Methylene-2,3,4,4-tetrame-2-Cyclopentenone			2.37			
	1860	Unknown Sesquiterpenol			2.06			
1679	1172	Trans-Alpha-Necrodol			6.56			
1696	1195	Alpha-Terpineol	0.11	0.29	0.29	4.86	0.13	0.44
1713	1167	Borneol	0.65	0.78		0.12	0.34	0.02
2168	1293	Thymol	36.72				68.83	0.08
2192	1316	Carvacrol	0.28			0.17	2.54	

**Table 3:** Antioxidant Activity Results (ABTS and DPPH methods). (Note 1: mean ± standard deviation) (Note 2: EO: Essential oil, OVV: *O*. *vulgare* subsp. *virens*, LPS: *L*. *pedunculata* subsp. *sampaioana*, LSL: *L*. *stoechas* subsp. *luisieri*, TM: *Th*. *mastichina*, TZS: *Th*. *zygis* subsp. *sylvestris*, MP: *M*. *× piperita*).

Code	ABTS		DPPH	
	mM TROLOX eq.	g TROLOX eq./g EO	mM TROLOX eq.	g TROLOX eq./g EO
OVV	76.45 ± 3.02	433.01 ± 17.10	25.15 ± 1.69	142.45 ± 9.57
LPS	3.84 ± 0.26	20.79 ± 1.41	2.17 ± 0.16	11.73 ± 0.87
LSL	24.06 ± 0.64	131.33 ± 3.47	33.91 ± 1.21	184.99 ± 6.58
TM	9.76 ± 0.41	54.19 ± 2.30	0.96 ± 0.03	5.31 ± 0.16
TZS	161.70 ± 0.15	864.20 ± 0.81	25.34 ± 1.08	135.42 ± 5.78
MP	4.83 ± 0.09	26.94 ± 0.51	3.83 ± 0.13	21.34 ± 0.74

**Table 4:** Statistical parameters from inhibition halo measurement (Note 1: x̅ = mean, s = standard deviation, Me = median, Max = maximum value, Min = minimum value, SEM = standard error) (Note 2: OVV: *O*. *vulgare* subsp. *virens*, LPS: *L*. *pedunculata* subsp. *sampaioana*, LSL: *L*. *stoechas* subsp. *luisieri*, TM: *Th*. *mastichina*, TZS: *Th*. *zygis* subsp. *sylvestris*, MP: *M*. *× piperita*).

EO	x̅	s	Me	Max	Min	SEM
*P. digitatum*						
OVV	31.33	3.21	30.00	35.00	29.00	1.86
LPS	20.17	3.25	20.00	23.50	17.00	1.88
LSL	30.67	1.15	30.00	32.00	30.00	0.67
TM	16.17	0.29	16.00	16.50	16.00	0.17
TZS	60.50	5.77	60.00	66.50	55.00	3.33
MP	19.33	3.21	18.00	23.00	17.00	1.86
*P. italicum*						
OVV	27.00	6.38	28.50	32.50	20.00	3.69
LPS	14.17	0.76	14.00	15.00	13.50	0.44
LSL	37.33	2.52	37.00	40.00	35.00	1.45
TM	13.67	1.26	13.50	15.00	12.50	0.73
TZS	54.33	2.93	55.50	56.50	51.00	1.69
MP	20.33	3.06	21.00	23.00	17.00	1.76

## Data Availability

The original contributions presented in this study are included in the article. Further inquiries can be directed to the corresponding author.
